# Thermal artefacts in two-photon solar cell experiments

**DOI:** 10.1038/s41467-018-07166-1

**Published:** 2019-02-27

**Authors:** Chris C. Phillips

**Affiliations:** Physics Department, Imperial College London, London, SW7 2AZ UK

**Keywords:** Solar cells, Nanophotonics and plasmonics

Asahi et al. recently reported^1^ record increases (ΔEQE) in the external quantum efficiency (EQE) of a heterojunction solar cell when it is illuminated with below-bandgap energy light. The EQE is the ratio of photocurrent electron flux to incident photon flux at zero bias. This ‘two-step photon up-conversion’ effect offers a way of breaking the 31% theoretical Shockley–Queisser solar cell efficiency limit^2^. However, the device transport is very temperature sensitive, and a 10 K temperature rise (see Fig. 3b, ref. ^1^) increases the photocurrent by about 30% on its own^1^. The below-bandgap light is continuous wave (CW) and intense (about 360 mW cm^−2^). Here it is argued that the observed photocurrent increase is due to sample heating, not direct photoexcitation.

Bandgap light (wavelength, *λ* of ∼780 nm) creates photocarrier pairs in the GaAs which are separated by the depletion field. It is claimed that photoelectrons accumulate in a long-lived intermediate state^3^ at a 220 meV high Al_0.3_Ga_0.7_As/GaAs heterojunction barrier in the depletion zone, before being photoexcited over it by the below bandgap (*λ* = 1300 nm) light and thus increasing the efficiency.

The best (ΔEQE = 30%) result came from a device measured in a solar simulator that also had a layer of InAs quantum dots (QDs) at the heterojunction^1^. However, a second measurement, using a 110 mW of CW (*λ* = 780 nm) laser instead of the simulator, saw the short-circuit current (*J*_sc_) increase from 6.6 to 7.2 mA cm^−2^, corresponding to a much lower ΔEQE of ∼0.95%. In other words, applying the 360 mW cm^−2^ of CW laser, with a wavelength of *λ* = 1300 nm, increased the photocurrent by a fraction of about 8.5%. This would correspond to a sample warming of <3 K.

The large (32 times) discrepancy between the two ΔEQE measurements is not discussed in the paper^1^, so one can only speculate on its origins. The simulator illumination intensity was about 2 mW cm^−2^ spectrally integrated, corresponding to about 29 µW cm^−2^ during the 350 nm wide EQE scan at 5 nm resolution (see Fig. 2a, b, ref. ^1^). The 1300 nm laser was therefore between 180 and 12,000 times brighter than the simulator was designed to work with. This could have both heated the sample, and blinded the simulator’s sensitive reference channel optical detectors with scattered laser light, causing it to over-report the ΔEQE. This would also explain why, somewhat surprisingly, a record ΔEQE (∼10%) was also seen in the control sample (see Fig. 2d, ref. ^1^) that had no QDs.

Concerning the photoexcitation over the barrier, the control sample result (ΔEQE approximately 10%) (see Fig. 2d, ref. ^1^) would have to be due to free-carrier absorption by the accumulated electrons. At these wavelengths, free-carrier absorption gives a cross section of about 6 × 10^−18^ cm^2^ per electron^4^, and the fact that the QD and control samples gave comprable ΔEQE results in the simulator would argue that the QD absorption cross section is similar. Taking the laser measurement for definiteness, the 110 mW cm^−2^ of bandgap light generates a photoelectron flux of about 4.1 × 10^15^ cm^−2^ s^−1^. Dividing this by the photon flux in the 320 mW cm^−2^ beam (*λ* of 1300 nm) would imply that 1.95 × 10^−3^ of the latter is involved in photoexciting electrons over the barrier. To get this absorption would need at least roughly 3.3 × 10^14^ cm^−2^ electrons to be trapped at the barrier.

This electron density is about 330 times higher than the *n*_s_ of around 10^12^ cm^−2^ density of the QDs. It is also roughly 3300 times higher than the electron density (*n*_s_ of around 1 × 10^11^ cm^−2^) that would be enough to generate a field discontinuity, Δ*E* proportional to *en*_s_*/ϵ*_0_*ϵ*_r_, that is large enough to screen out^5^ the roughly 1.4 × 10^6^ V m^−1^ depletion field that is needed to trap the electrons. This argues that the ΔEQE effect cannot be explained by photoexcitation at the barrier.

Also reported was a roughly 2 mV increase in the roughly 700 mV open-circuit voltage (*V*_oc_) when the *λ* equals 1300 nm light was applied (Fig. 7 in ref. ^1^). By comparing this with the 20 mV approximate drop in *V*_oc_ seen when the device is warmed by 10 K, it is argued that the light-induced rise in *V*_oc_ must be non-thermal, because warming a normal p–n junction increases the diode leakage current and therefore reduces *V*_oc_, whereas increasing the photocurrent will always increase *V*_oc_. Firstly, these statements only apply to an ideal Shockley p–n junction, with a drift-diffusion^6^ dark current and a constant photocurrent. Neither conditions apply to Asahi et al.’s heterostructure, where both photocurrent and the leakage currents increase strongly with temperature^1^. They will be influencing *V*_oc_ in opposite directions and in practice the direction of change of *V*_oc_ will depend in a complex way on bandgap changes and on experimental parameters, such as the bandgap illumination intensity, so the observed sign of he change cannot be taken as evidence that it is caused by photoexcitation.

Secondly, there is the problem of experimental drift. The quoted 0.4 mV accuracy on *V*_oc_ (Fig. 7b error bars) translates into a *J*_sc_ uncertainty of about 2.5 × 10^−5^ A cm^−2^. This is only 1/270th of the ∼7 mA cm^−2^ photocurrent. Furthermore, the fact that Fig. 7a inset plot shows no data points or experimental noise implies a *V*_oc_ error that is less than the width of the lines in the graph. Measuring off the plot this is about 0.1 mV, i.e. about four times less than the error bars in Fig. 7b, so Fig. 7a, b are not compatible.

To summarise, one has to conclude that Fig. 7 data could only be taken at face value if experimental parameters, such as the excition laser intensity and the effects of ambient light fluctuations were all controlled to better than one part in about 1000. This would be a formidable experimental achievement.

Finally, Asahi et al. point out that their ΔEQE signals are “approximately two orders of magnitude greater than previously reported”, and cite a number of related experiments. Many of these, and others in the literature, describe CW measurements that are also susceptible to these thermal artefacts.

ΔEQE signals with a thermal origin have much slower response times than genuine electronic ones. They can be eliminated by intensity modulating the excitation laser(s) and checking that the ΔEQE signal is independent of the modulation frequency^5^. The claims of Asahi et al. are incorrect becaue they have failed to demonstrate this. The purpose of this correspondence is to urge the experimental community to apply this simple experimental test in future work.

## Data Availability

The data are available from the author on request.
